# The Membranotropic Peptide *gH625* to Combat Mixed *Candida albicans*/*Klebsiella pneumoniae* Biofilm: Correlation between In Vitro Anti-Biofilm Activity and In Vivo Antimicrobial Protection

**DOI:** 10.3390/jof7010026

**Published:** 2021-01-05

**Authors:** Angela Maione, Elisabetta de Alteriis, Federica Carraturo, Stefania Galdiero, Annarita Falanga, Marco Guida, Anna Di Cosmo, Valeria Maselli, Emilia Galdiero

**Affiliations:** 1 Department of Biology, University of Naples Federico II, Via Cinthia 26, 80126 Naples, Italy; angela.maione@unina.it (A.M.); dealteri@unina.it (E.d.A.); federica.carraturo@unina.it (F.C.); marco.guida@unina.it (M.G.); dicosmo@unina.it (A.D.C.); 2 Department of Pharmacy, School of Medicine, University of Naples Federico II, Via Domenico Montesano 49, 80131 Naples, Italy; sgaldier@unina.it; 3 Department of Agricultural Science, University of Naples Federico II, Via dell’Università 100, 80055 Portici, Naples, Italy; annarita.falanga@unina.it

**Keywords:** *Galleria mellonella*, polymicrobial biofilm, experimental method in vivo

## Abstract

The antibiofilm activity of a gH625 analogue was investigated to determine the in vitro inhibition and eradication of a dual-species biofilm of *Candida albicans* and *Klebsiella pneumoniae*, two leading opportunistic pathogens responsible for several resistant infections. The possibility of effectively exploiting this peptide as an alternative anti-biofilm strategy in vivo was assessed by the investigation of its efficacy on the *Galleria mellonella* larvae model. Results on larvae survival demonstrate a prophylactic efficacy of the peptide towards the infection of each single microorganism but mainly towards the co-infection. The expression of biofilm-related genes in vivo showed a possible synergy in virulence when these two species co-exist in the host, which was effectively prevented by the peptide. These findings provide novel insights into the treatment of medically relevant bacterial–fungal interaction.

## 1. Introduction

Microorganisms rarely exist as single-species planktonic forms, and the biofilm mode of growth is the most common lifestyle adopted. Biofilms can be defined as biotic and abiotic surface-associated, structured microorganism communities embedded in an extracellular polysaccharide matrix. Living in a biofilm provides protection in a stressful environment where mechanical stress, desiccation, and biocides are frequent threats.

Progress in biofilm research has highlighted that these communities are rarely composed of a single-species microorganism, but mainly exist as complex, diverse, and heterogeneous structures. In fact, multiple species (fungi, bacteria, and viruses) frequently exist together in complex polymicrobial biofilm communities attached to sites where they compete for space and nutrients [1,2,3]. Moreover, polymicrobial biofilms are likely to influence disease severity by promoting intensified pathogenic phenotypes, including increased resistance to both host defense and antimicrobial therapies. Despite their clinical significance, polymicrobial biofilm infections continue to be largely understudied [4].

*Candida albicans* and *Klebsiella pneumoniae* are important pathogens causing a wide variety of infections. They possess the ability to co-exist as complex polymicrobial biofilms within the human host [5]. *C. albicans* is the predominant fungus frequently present in hospital infections with significant morbidity and mortality rates; unfortunately, it is difficult to prevent, diagnose and treat. It is an opportunistic pathogen, which is a major cause of invasive fungal disease, principally in immunocompromised individuals, such as patients with organ transplantation and HIV infection or patients undergoing chemotherapy. The superficial mucosal and dermal infections caused by *C. albicans* can be disseminated to bloodstream infections with a mortality rate higher than 40% [6].

*Klebsiella pneumoniae*, the most common carbapenem-resistant member of the *Enterobacteriaceae* family, has emerged as an important opportunistic pathogen, mostly causing nosocomial infections associated with mortality rates up to 50% [7]. *K. pneumoniae* is responsible of infections both in human gastrointestinal tract and lung environments, and has a high propensity to form mono- and polymicrobial biofilms with consequent treatment difficulties [8].

Mixed biofilms of *Candida* with different bacteria, including *K. pneumoniae*, are present on implanted devices such as intravascular or urinary catheters, as well as in the oral environment [2].

Notwithstanding its clinical importance, information on mixed biofilms of *Candida/Klebsiella* is relatively scarce [9].

The potential use of antimicrobial peptides (AMPs) as a valid alternative to conventional antibiotics has been acknowledged and widely studied. In fact, their fast and strong antimicrobial activity, their antibiofilm action, and their reduced induction of resistance compared to conventional antibiotics make AMPs relevant compounds for controlling infections due to multi-drug resistant microorganisms embedded in a biofilm [10,11,12]. Among AMPs, there is a particularly relevant class of peptides, known as membranotropic peptides, which, apart from their eventual antimicrobial activity, present a high ability to disrupt the biofilm and thus may have an action both in the inhibition and in the eradication of the biofilm [13].

In this study, we evaluated the anti-biofilm activity of an analogue of the membranotropic peptide gH625, namely gH625-M, on a dual-species *C. albicans*/*K. pneumoniae* biofilm. Peptide gH625-M (HGLASTLTRWAHYNALIRAFGGGKKKK) is derived from gH625 (HGLASTLTRWAHYNALIRAF) and presents a C-terminal lysine sequence conjugated to the gH625 peptide by a glycine linker; the lysine functions to enhance the interaction with the negatively charged surfaces of bacterial biofilms and to enhance solubility. The glycine linker between the gH625 sequence and the lysine residues provides conformational flexibility to the peptide. gH625-M was shown to have activity on polymicrobial biofilms of *Candida tropicalis* and *Serratia marcescens* or *Staphylococcus aureus* [14] and on biofilms derived from *C. albicans* persister cells [15]. Therefore, the peptide was selected as a good candidate to evaluate the antibiofilm activity in vitro on a static biofilm of *C. albicans*/*K. pneumoniae*.

Since in vivo studies are crucial for the evaluation of the antimicrobial activities of new therapeutic agents and their modulatory effects on immune response, the larvae of the wax moth *G. mellonella* were used. In particular, *G. mellonella* is frequently exploited as an alternative to the murine model for studying microbial infections, because it is a simple, cheap and fast method, and implies fewer ethical concerns compared to the use of vertebrate models. As a matter of fact, several other studies have recently used *G. mellonella* to investigate the in vivo activity of antimicrobial agents against pathogenic microorganisms, including bacteria and fungi [16].

Here, we investigated for the first time, the correlation between the susceptibility profile shown by gH625-M in vitro and its antimicrobial efficacy in vivo. Therefore, the infection in the *G. mellonella* larva model was evaluated through the impact of gH625-M on the survival rate and on the immune response (galiomycin) of the larvae [17]. Furthermore, the expression of biofilm related genes of *C. albicans* (*HWP1*, *ALS3*) [18], and *K. pneumoniae* (*luxS*, *mrkA*) in *G. mellonella* larvae was investigated [19,20].

## 2. Materials and Methods

### 2.1. Peptide Synthesis

Peptide gH625-M (HGLASTLTRWAHYNALIRAFGGGKKKK) was synthetized by the Fmoc-solid-phase method [21]. Briefly, all amino acids were protected at their amino terminus with the Fmoc (9-fluorenylmethoxycarbonyl) group and coupled to the growing chain after activation of the carboxylic acid group. Consecutive cycles of amino deprotection (30% piperidine in dimethylformamide, for 10 min, twice) and coupling were performed to obtain the desired sequence. In particular, the first coupling was performed with four equivalents (equiv) of amino acid and four equiv DIC (Diisopropyl carbodiimide); while from the second coupling, we used four equiv amino acid, four equiv HATU (O-(7-azabenzotriazol-1-yl)-1,1,3,3-tetramethyluroniumhexafluorophosphate) and eight equiv DIPEA (diisopropylethylamine): the synthesis was performed using a rink amide resin MBHA (4-methyl-benzhydrylamine-resin, 0.44 mmol/g). Side chain deprotection and cleavage of the peptide from the resin was achieved using an acid solution (95% *v/v* of TFA, trifluoroacetic acid). The peptide was precipitated in cold ethylic ether and the crude peptide was analyzed by HPLC–MS using a gradient of acetonitrile (0.1% TFA) in water (0.1% TFA) from 20 to 80% in 15 min. The purified peptide was obtained with a good yield (approximatively 60%) and its identity was confirmed using a LTQ-XL Thermo Scientific linear ion trap mass spectrometer.

### 2.2. Microorganisms Culture and Biofilm Formation

*Candida albicans* ATCC 90028 and *Klebsiella pneumoniae* ATCC 10031 were selected as pathogen representative of fungus and Gram-negative bacteria forming biofilm in hospital environments. *C. albicans* ATCC 90028 was subcultured into Tryptone Soya Broth (TSB) (Oxoid) medium with 1% of glucose and propagated in Sabouraud dextrose agar (1% yeast extract, 1% peptone, 4% glucose, 1% agar) as described previously [14]. *K. pneumoniae* ATCC 10031 was grown in Tryptone Soya Broth (TSB and maintained in Tryptone Soya Agar (TSA) at −80 °C.

Biofilm formation was carried out on microtiter plates, as previously described [14], with minor modifications. Briefly, for single species biofilms. *C. albicans* and *K. pneumoniae* suspensions were adjusted to 10^6^ colony-forming units (CFU) mL^−1^ in fresh TSB with 0.1% glucose and TSB, respectively. In the case of the dual species biofilm, microorganism suspensions were mixed in TSB with 0.1% glucose (1:1). Aliquots (100 μL) of these suspensions (single or mixed) were added to the wells of sterile flat-bottom 96-well microtiter test plates to allow single or mixed biofilm formation and incubated for 24 or 48 h at 37 °C. Then, the wells were washed with PBS in order to remove planktonic and sessile cells weakly attached to the surface. Quantification of biofilm biomass was carried out with crystal violet (CV) staining [22].

Experimental conditions were run in triplicate. Results are presented as mean values from at least four independent experiments.

### 2.3. Minimum Inhibition Concentration (MIC)

The concentration of the peptide that inhibited 80% microbial growth (MIC_80_) was determined by the microdilution method according to Clinical Laboratory Standards Institute guidelines [23]. Briefly, 100 μL of TSB with 1% glucose and TSB containing strain of *C. albicans* or *K. pneumoniae*, respectively (1 × 10^6^ CFU/mL), was introduced into each well of 96-well microplate with different concentrations of gH625-M (2.5 μM–50 μM) and incubated for 24 h at 37 °C. The growth of each strain was measured at 590 nm wavelength with a plate reader (SYNERGY H4 BioTek).

### 2.4. Inhibition and Eradication Biofilm Assays

The inhibition activity of gH625-M on mono- and polymicrobial biofilm formation and the eradication activity against preformed biofilm were evaluated by using sub-MIC concentrations of the peptide ranging from 2.5 to 50 μM.

Briefly, for the inhibition assay, peptide was added together with the standardized inoculum in each well of a 96-well polystyrene microtiter plate and incubated at 37 °C for 24 h. Non-adherent microorganisms were removed by washing twice with 200 μL sterile PBS and adherent cells were fixed by incubation for 1 h at 60 °C and stained for 5 min at room temperature with 100 μL 1% crystal violet solution. Wells were then rinsed with distilled water and dried at 37 °C for 30 min. Biofilms were de-stained by treatment with 100 μL 33% glacial acetic acid for 15 min and OD_570_ measured.

The eradication activity of the peptide was evaluated exposing 24 h mono- and polymicrobial biofilms for additional 24 h to different sub-MIC concentrations of peptide and quantified the biomass by crystal violet assay as previously described. The percentages of inhibition or eradication were calculated as biofilm reduction % = OD_570_ control − OD_570_ sample/OD_570_ control × 100, where OD_570_ control and sample were the biomass formed in the absence and in the presence of the peptide, respectively. All tests were performed in triplicate in three independent experiments.

### 2.5. Galleria Mellonella Survival Assay

To determinate the in vivo effects of gH625-M, a *G. mellonella* survival assay was performed as described previously [24,25]. In brief, larvae of 250–300 mg each were used for each treatment (20 for each group). They were chosen to have clear color and a lack of spots and/or dark pigments on their cuticle. The experiments were performed in triplicate.

Larvae were cleaned by an alcohol swab prior to injection. Larvae were injected directly into the hemocoel with 10 μL *C. albicans* and/or *K. pneumoniae* suspensions prepared in PBS at a concentration of 1 × 10^6^ CFU/larvae/pathogen (1 × 10^6^ CFU/larvae total for co-infection), using a 50 μL microsyringe via the last left proleg. An aliquot of 10 μL of 50 μM gH625-M was delivered behind the last proleg on the opposite side of the pathogen injection site either 2 h pre-infection (for prevention experiments) or 2 h post-infection (for treatment experiments). One group of untreated larvae served as a blank control group (intact larvae), one group received 10 μL of PBS solution per leg and one group was injected with 10 μL of 50 μM gH625-M in one leg and 10 μL PBS in the other, in order to assess peptide toxicity.

Larvae were then incubated at 35 °C in plastic containers provided with a perforated lid and monitored daily for survival for 4 days. A larva was considered dead when it displayed no response to touch.

### 2.6. Fungal/Bacterial Burden

Larvae were inoculated with *C. albicans* or *K. pneumoniae* or co-infected with the two, at concentration of 1 × 10^6^ CFU/larvae/pathogen or 1 × 10^6^ CFU/larvae total for co-infection. The peptide was administered before or after infection/co-infection, as described in the previous paragraph. The infected models were incubated at 30 °C, and after 24 h of infection, two larvae were randomly selected and washed in 70% ethanol. Larvae were cut into small pieces using a sterile scalpel and added to falcon tubes containing 1 mL of PBS vortexed and 100 µL of each sample was collected and serially diluted. The dilutions were plated and incubated 48 h at 30 °C and colony forming units (CFU) of *C. albicans* and *K. pneumoniae* were counted on Sabouraud dextrose agar plus 20 μg mL^−1^ cloramphenicol and TSA plus 1 μg mL^−1^ caspofungin, respectively. The experiments were performed in triplicate.

### 2.7. RNA Extraction and Gene Expression Analysis

Larvae were infected, and RNA was extracted at 4 and 24 h post-treatment.

Therefore, three live larvae from each experimental group (4 and 24 h post-treatment) were snap-frozen in liquid nitrogen and ground to a powder by mortar and pestle in TRIzol (Invitrogen, Paisley, UK). The samples were further homogenized using a TissueLyser II (Qiagen, Valencia, CA, USA) and steal beads of 5 mm diameter (Qiagen, Valencia, CA, USA). RNA was extracted with RNeasy minikit (Qiagen, Valencia, CA, USA), following the manufacturer’s protocol. The quality and amount of purified RNA were analyzed spectrophotometrically with Nanodrop2000 (Thermo Scientific Inc., Waltham, MA, USA). 1000 ng of RNA was reverse transcribed with the QuantiTect Reverse Transcription Kit (Qiagen, Valencia, CA, USA), used as described by the manufacturer. Afterwards, Real-Time PCR was performed using the QuantiTect SYBR Green PCR Kit (Qiagen, Valencia, CA, USA) in a final volume of 25 μL, with 100 ng of cDNA, 1 μM of each primer, 12.5 μL of QuantiFast SYBR Green PCR Master Mix (2×). PCR cycling profile consisted of a cycle at 95 °C for 5 min, 40 two-step cycles at 95 °C for 15 s, at 60 °C for 60 s. Quantitative RT-PCR analysis was conducted using the 2^(−ΔΔC(T))^ method [26]. RT-PCR was performed in a Rotor-Gene Q cycler (Qiagen, Valencia, CA, USA). All primers used for quantitative PCR (qPCR) studies are shown in Table 1. At the end of each test, a melting curve analysis was done (plate read every 0.5 °C from 55 to 95 °C) to determine the formation of the specific products. Each sample was tested and run in duplicate. No-template controls were included.

mRNA levels in the different treatments were compared by ANCOVA (analysis of covariance). The control and the treatment groups in various assays were compared and analyzed using a Wilcoxon two group test and data with *p*-values < 0.05 were considered statistically significant [27].

Transcriptional activation is represented by the RNA fold change of the expression; for the *galiomycin* gene evaluation in *G*. *mellonella* actin was used as housekeeping gene, for the *luxS* and *mrkA* genes in *K. pneumoniae* 16S was used as housekeeping gene, and for *HWP1* and *ALS3* genes in *C. albicans* actin was used as housekeeping gene.

### 2.8. Scanning Electron Microscopy (SEM)

The 48 h polymicrobial biofilm was formed in multi-well plates as described above. The slides were prepared for scanning electron microscopy (SEM) using a previously published protocol [14]. Briefly, the slides were placed in 3% glutaraldehyde at 4 °C, then washed with PBS and post-fixed in 1% aqueous solution of osmium for 90 min at room temperature. Then, samples were dehydrated in a series of graded alcohols, dried to the critical drying point, and finally coated with gold. Specimens were evaluated with a scanning electron microscope (QUANTA 200 ESEM FEI Europe Company, Eindhoven, The Netherlands).

### 2.9. Statistical Analysis

Statistical analyses were performed using Microsoft^®^ Excel 2016/XLSTAT©-Pro (version 7.2, Addinsoft, Inc., Brooklyn, NY, USA). Error bars in the graphs represent standard error of the mean (SEM and gene expression analysis) or standard deviations (SD, for biomass in mono- and polymicrobial biofilms, inhibition and eradication biofilm assay, CFU assay).

In the *G. mellonella* model of infection, the survival curves were plotted using Kaplan–Meier method and log-rank test. In all other assays, Tukey test was used to compare the means within the same set of experiments and ANOVA to consider the differences among the groups. A *p*-value of <0.05 was considered statistically significant.

## 3. Results

**Figure 1:**
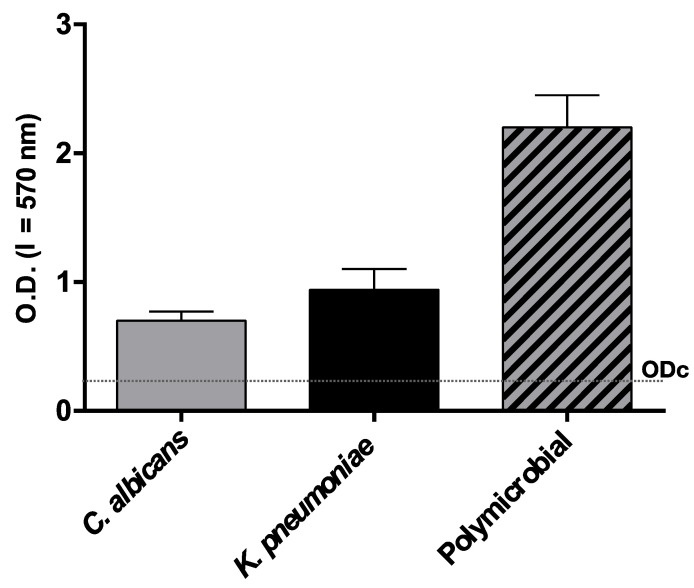
Biomass in mono- *(K. pneumoniae* and *C. albicans)* and polymicrobial biofilms (*n* = 3, mean ± SD) quantified by crystal violet staining and expressed as OD_570_. The dotted line corresponds to ODc, that is the cut-off value, defined as three standard deviations above the mean OD of the negative control. Negative (OD ≤ ODc), weak (ODc ≤ OD ≤ 2 ODc), moderate (2 ODc < OD ≤ 4 ODc), and strong biofilm production (4 ODc < OD), according to Stepanovich [22].

**Figure 2:**
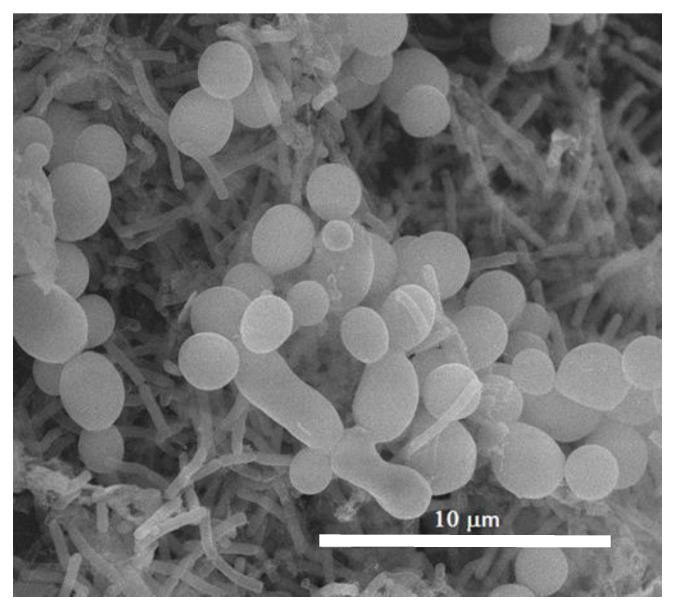
SEM observation of the 48 h dual species biofilm of *C. albicans* and *K. pneumoniae*. Scale bar = 10 µm.

Both the examined strains were able to form single- and dual-species biofilm in vitro (Figure 1) under the experimental conditions adopted. In particular, the single biofilm production was weak for both species and moderate for dual species. The biomass of the dual species biofilm was even higher than the sum of the single species biofilm biomass, which likely indicated a synergism between the two species forming the biofilm. The analysis of the SEM images clearly showed the strict interconnection between the two species in the mixed biofilm (Figure 2). The co-existence of *C. albicans* and *K. pneumoniae* was clearly shown in the SEM image (Figure 2) and also confirmed by cell count: 4.1 × 10^8^ ± 0.2 (SD) and 4.6 × 10^8^ ± 0.4 (SD), respectively.

The peptide gH625-M is an analogue of gH625, a peptide, which proved to be very effective in crossing membrane bilayers [15,28]. *In vitro*, gH625-M showed weak anticandidal activity with a MIC_80_ > 50 µM, which was in agreement with our previous studies [15]. Similarly, the MIC_80_ value of *K. pneumoniae* was higher than 50 µM, indicating a relatively scarce antibacterial activity of the peptide.

**Figure 3:**
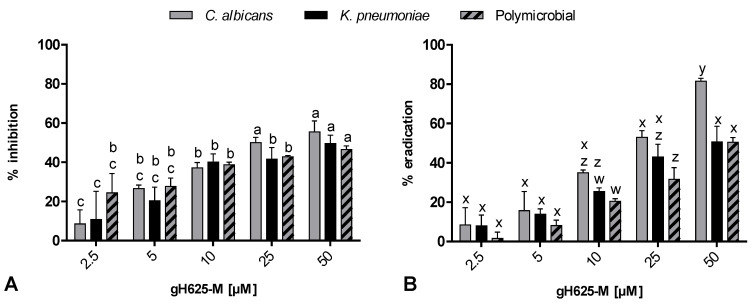
Action of increasing gH625-M concentrations on inhibition (**A**) and eradication (**B**) of mono- and polymicrobial biofilms. Quantification of the residual biofilm biomass was performed by crystal violet staining. Data with different letters (a–c; w–z) are significantly different (Tukey’s, *p* < 0.05).

Interestingly, gH625-M was able to inhibit the formation of the biofilm, as well as to eradicate it (Figure 3A,B). After treatment with sub-MIC doses of gH625-M, the formation of mono- and polymicrobial biofilm was inhibited significantly (Figure 3A).

Figure 3B showed the eradication effect of gH625-M on preformed mono- and polymicrobial biofilm (48 h old). At 50 µM, gH625-M reduced the mono-preformed biofilms of *C. albicans* and *K. pneumoniae* of 80% and 50%, respectively, and the mixed biofilm of 50%.

**Figure 4:**
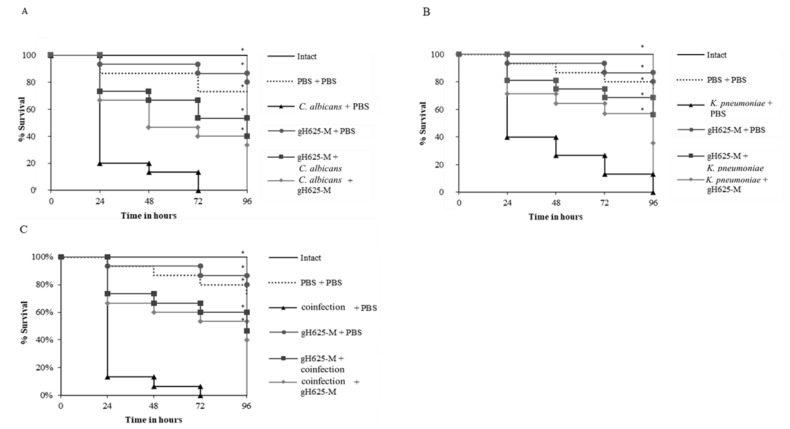
Kaplan–Meier plots of survival curves of *G. mellonella* larvae infected with *C. albicans* (**A**), *K. pneumoniae* (**B**), and co-infected with *C. albicans* + *K. pneumoniae* (**C**). In all panels, survival curves of larvae are shown. All groups were treated with 50 μM gH625-M before or after infection/co-infection are reported. All groups were compared with control (infected or co-infected larvae). In all panels, survival curves of intact larvae, larvae injected with PBS, larvae injected with gH625 alone are reported. * represents *p*-value < 0.001 (log-rank test).

The in vivo antimicrobial activity of gH625-M was evaluated using *G. mellonella* larvae infected with *C. albicans* and *K. pneumoniae* isolates alone or mixed as shown in Figure 4.

Larval survival assay indicated that groups of larvae injected with PBS alone and gH625-M alone presented about 80% survival up to 96 h of observation with respect to intact larvae, indicating that gH625-M was not toxic for the larvae. Survival of larvae infected with *C. albicans* or *K. pneumoniae* (Figure 4A,B) was only 20% and 40% after 24 h, with 100% mortality observed after at 72 and 96 h, respectively.

To determine whether gH625-M had an effect in vivo, *G. mellonella* larvae were treated with gH625-M at 50 µM before or after the infection with each of the two species (Figure 4A,B) and before or after co-infection with the two (Figure 4C).

Both pre- and post-infection treatments showed significantly higher survival rates. In particular, the survival of larvae treated with gH625-M before infection with *C. albicans* was about 70%, and for *K. pneumoniae* was 80% after 24 h, preserving about 50% and 70% survival respectively at the end of the observation period. Moreover, the survival of the larvae treated with gH625-M after the infection with *C. albicans* or *K. pneumoniae* was significant, resulting in higher than 60% at 24 h, and 40% at 72 h of the experiment for both the microorganisms.

These results indicate that when gH625-M was administered before the infection, it was more effective compared to when administered after infection for both microorganisms. However, the increased survival rate of infected *Galleria* further confirms the significant activity of gH625-M.

In Figure 4C, survival of larvae co-infected with both *C. albicans* and *K. pneumoniae* is reported. Compared to single infection, mortality was enhanced, being 90% and 100% after 24 and 72 h. For co-infections, the administration of gH625-M greatly improved larvae survival both when given before and after the co-infection, with a slightly better prophylactic effect.

**Figure 5:**
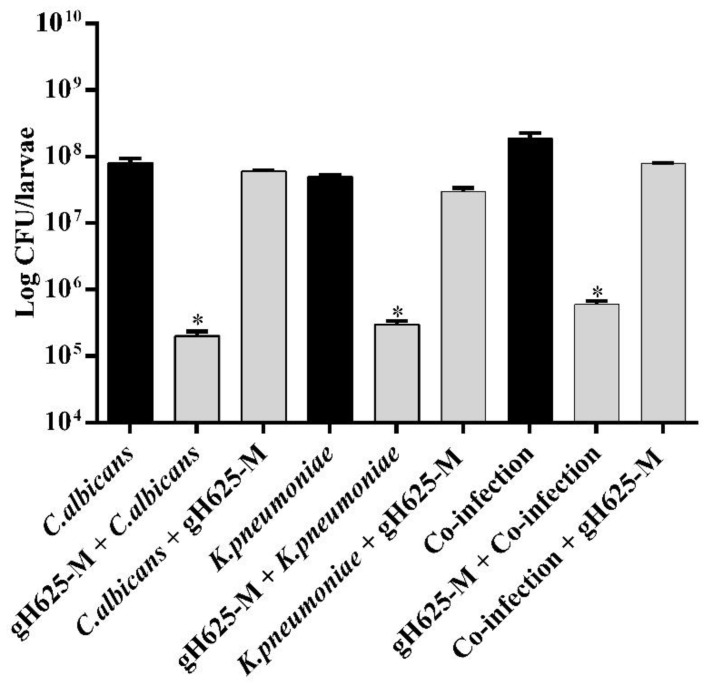
Effect pre- and post-treatment with gH625-M on bacterial/fungal burden in *G. mellonella* infected with *C. albicans* or *K. pneumoniae* and co-infected with *C. albicans* + *K. pneumoniae*. The peptide was administered before or after infection/co-infection. * indicates that the differences vs. larvae injected with microorganisms alone or together is statistically significant (*t*-test; *p* < 0.05). Error bars represent the SD.

To detect the effect of gH625-M on the fungal and bacterial burden of infected larvae, a burden analysis was performed (Figure 5).

There was a significant decrease in the microbial burden for the gH625-M pre-treated groups and only a slight decrease for the gH625-M post-treated groups. These results corroborated the protective action of gH625-M towards infection and co-infection, as already seen in the analysis of survival curves.

The expression of the galiomycin peptide-encoding gene is associated with the immune response of *G*. *mellonella*. Galiomycin is an antimicrobial peptide playing a major role in innate immunity, showing broad-spectrum microbicidal activity and specificity to *G. mellonella*. To evaluate the insect humoral response after infection with and co-infection each of the two microorganisms and co-infection, as well as the role of gH625-M on infection and co-infection we evaluated the expression of the *galiomycin* peptide-encoding gene.

**Figure 6:**
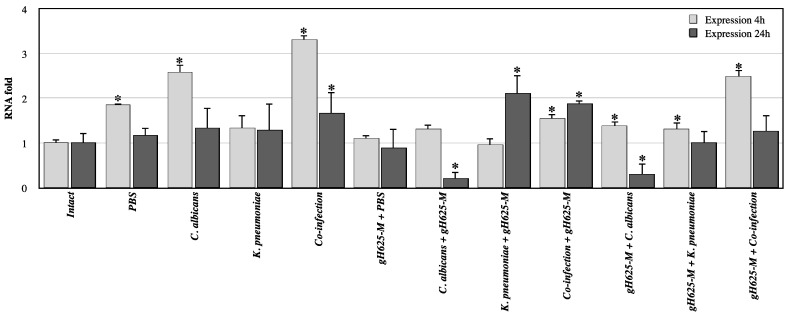
Relative mRNA expression levels of galiomycin gene in *G. mellonella* larvae infected with *C. albicans* and *K. pneumoniae* alone or together and pre-treated or post-treated with gH625-M, at 4 and 24 h, measured using real-time PCR analysis and calculated by the 2^(−ΔΔC(T))^ method. Actin gene was used as housekeeping. Each sample was tested and run in duplicate. No-template controls were included. * asterisk indicates that the difference vs. intact larvae expression is statistically significant (Wilcoxon two group test, *p* < 0.05). Error bars represent the SEM.

Figure 6 reports expression levels of *galiomycin* gene in *G. mellonella* larvae after 4 and 24 h after infection with each species, after co-infection with the two species and after treatment with the peptide. Analysis by real-time quantitative PCR showed that the levels of galiomycin were higher in insects infected after 4 h with *C. albicans* alone (*p* < 0.05) and co-infected with *C. albicans* and with *K. pneumoniae* (*p* < 0.05) than those found in insects infected with *K. pneumoniae* alone. Instead, the expression levels of the same gene after 24 h from the infection did not significantly differ among the insects infected with both microorganisms. In this context, it could be reasonable to explore some other markers able to evidence the biofilm-associated damage in infected larvae, or lack thereof in gH625M-treated larvae.

Analysis of the expression of the selected genes revealed that the use of gH625-M before and after the infection, and the co-infection, significantly affects expression, decreasing the level of *galiomycin* compared to the corresponding intact and not treated samples. Data also show that the treatment with gH625-M produces an inhibition of galiomycin gene expression.

**Figure 7:**
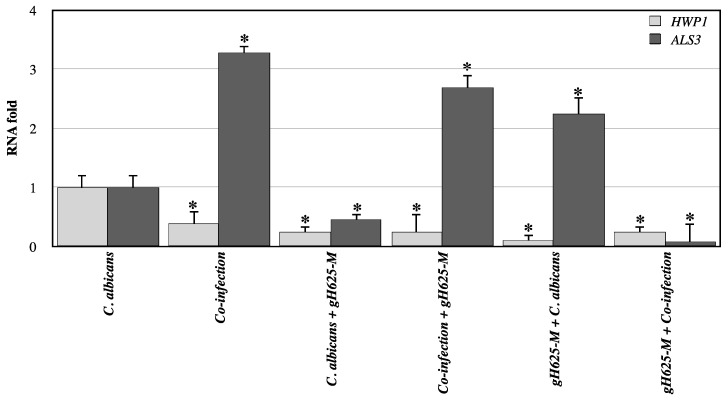
Relative mRNA expression levels of *C. albicans* virulence genes (*HWP1* and *ALS3*) in *G. mellonella* larvae at 24 h, measured using real-time PCR analysis and calculated by the 2^(−ΔΔC(T))^ method. Actin gene was used as housekeeping. Each sample was tested and run in duplicate. No-template controls were included. * asterisk indicates that the difference vs. expression of larvae treated with *C. albicans* only is statistically significant (Wilcoxon two group test, *p* < 0.05). Error bars represent the SEM.

In Figure 7, levels of hyphal-specific and biofilm-related genes in *C. albicans* in the absence and presence of gH625-M at 24 h were quantified by real-time PCR. Hyphal specific gene, *HWP1* was downregulated in all cases showing hyphal structure formation failure. Biofilm-related gene *ALS3* was significantly downregulated in the presence of gH625-M 2 h after infection with *C. albicans* and in polymicrobial treated with gH625-M two hours before the infection. It was upregulated in larvae with only *C. albicans* infection pre-treated and in polymicrobial biofilm untreated or post-treated with gH625-M.

**Figure 8:**
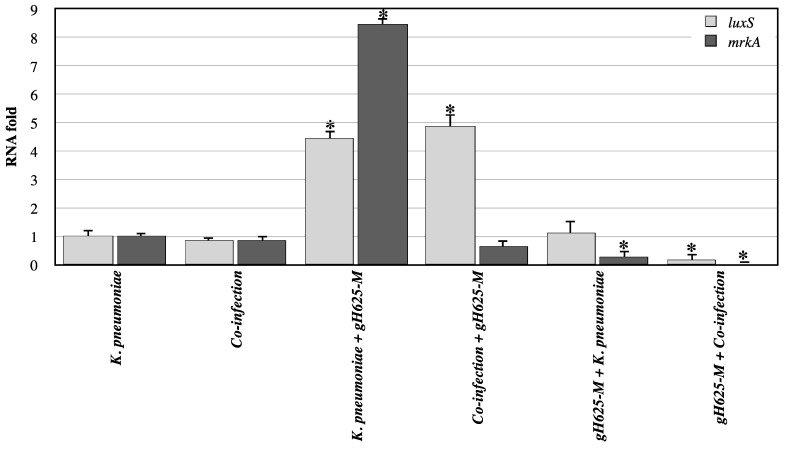
Relative mRNA expression levels of *K. pneumoniae* virulence genes (*luxS* and *mrkA*) in *G. mellonella* larvae at 24 h, measured using real-time PCR analysis and calculated by the 2^(−ΔΔC(T))^ method. 16S gene was used as housekeeping. Each sample was tested and run in duplicate. No-template controls were included. * asterisk indicates that the difference vs. expression of larvae treated with *K. pneumoniae* only is statistically significant (Wilcoxon two group test *p* < 0.05). Error bars represent the SEM.

The relative expressions of *luxS* and *mrkA* genes were evaluated in *G. mellonella* infected with *K. pneumoniae* alone or in combination with *C. albicans* (Figure 8). The *luxS* gene encodes the AI-2 proteins of quorum sensing that play an important role in biofilm formation in Gram-negative bacteria such as *K. pneumoniae*, while *mrkA* gene (type 3 fimbriae) is a virulence -related gene detected in *K. pneumoniae;* both have critical roles in biofilm formation and antibiotic resistance.

It is interesting to observe that in co-infections, when the peptide was administered before, both *mrkA* and *luxS* genes were significantly down-regulated compared to untreated co-infections. The effect of pre-treatment was also observed in the case of *K. pneumoniae* infection. In contrast, both *mrkA* and *luxS* genes were upregulated when gH625-M was administered after infection and co-infection.

## 4. Discussion

Infections associated with polymicrobial fungal/bacterial biofilms represent a huge challenge due to intrinsic heterogeneity of these consortia, the low susceptibility to traditional drugs, as well as the high toxicity of many common antifungals. In this context, the development of novel strategies to combat polymicrobial biofilms of pathogen species is of great relevance.

In this paper, we focused our attention on the dual-species biofilms formed by *C. albicans* and *K. pneumoniae*, with *C. albicans* being the most common fungal opportunistic pathogen and *K. pneumoniae* being recognized in the group of ‘ESKAPE’ (*Enterococcus faecium*, *Staphylococcus aureus*, *Klebsiella pneumoniae*, *Acinetobacter baumannii*, *Pseudomonas aeruginosa*, *Enterobacter* spp.) pathogens, which ‘escape’ from the action of several antibiotics [29].

Previous work has demonstrated that the membranotropic peptide gH625-M was very effective against *C. albicans* biofilm, also when the biofilm was developed from persister cells [15]. In the present paper, we explored the efficacy of the peptide towards the dual-species *Candida*/*Klebsiella* biofilm, showing its ability of both inhibiting and eradicating in vitro the biofilm at sub-MIC concentrations. The low antimicrobial activity of the peptide used in this work towards *C. albicans* and *K. pneumoniae* was very low and was similar to that of other previously tested membranotropic peptides [14,15]; nevertheless, these characteristics may represent an advantageous property in the prevention of the possible development of resistances in the entire microbial consortium, a feature typical of several compounds proposed as anti-biofilms molecules [11]. The mechanism of action of gH625-M against *C. albicans* monospecies biofilm, as shown by a CSLM analysis previously reported [15], is initially directed towards the esopolymeric matrix of the biofilm and then penetrates across cell membranes, producing a local and temporary destabilization of membranes, which nonetheless has scarce effects on cell viability. The results obtained in the present paper, with the scarce antimicrobial activity (MIC values > 50 uM) exhibited in the case of both fungal (*Candida*) and bacterial (*Klebsiella*) cells, further support the hypothesis that the de-structuring property is presumably at the basis of the inhibiting and eradicating efficacy of the peptide also in the case of the *Candida*/*Klebsiella* biofilm. Interestingly, the dual-species biofilm is characterized by a strict interconnection established between the two species, thus the ability to disrupt the structure of the biofilm is of even greater relevance.

*Candida/Klebsiella* biofilm-associated infections have been reported in mammalian hosts; nonetheless, the description of the interactions between *Candida* spp. and *K. pneumoniae* in mixed biofilms has been limited to few observations, derived from in vitro models [9]. Our results show an increase in the total biomass of the polymicrobial biofilm in vitro compared to the sum of the single species biofilm, suggesting a possible synergy between the two species.

In vivo studies are crucial for the evaluation of the efficacy of new therapeutic agents and their modulatory effects on the host immune response, which makes it necessary to evaluate outcomes in animal models. In this study, we used the larvae of the wax moth *G. mellonella* as an in vivo model, for its well-known advantages as an alternative to vertebrate models. Although *G. mellonella* does not replace the vertebrate model, it can be exploited as a screening step between in vitro and in vivo evaluations [16].

One of the main outcomes was derived from the analysis of the survival curves after fungal/bacterial infection in the larvae [30]. Our results clearly show that the killing of *G. mellonella* larvae by infection of the two pathogens together is greater than the sum of killing with each pathogen alone; this pattern suggests a synergistic pathogen–pathogen interaction or a change in host–pathogen interactions that is characterized by increased host susceptibility to one or both of the pathogens.

The analysis of the survival curves in experiments with gH625-M, showed that the peptide functions in vivo both as a prophylactic and therapeutic agent towards both single infections and co-infections, with a higher efficacy in prophylaxis as revealed also with the fungal burden analysis.

The actual interaction established in vivo between the two species examined and the host, as well as the action of the peptide, was further investigated in the larvae model through the analysis of the transcriptional profiles of both biofilm-associated genes and gene associated with the larvae innate immune response. Following infection, the expression of the biofilm-associated genes of the two species examined was enhanced in the larvae, suggesting the occurrence of a biofilm-like interaction in the animal, which was concomitant to the overexpression of the *galiomycin* gene indicating an activation of the larvae immune response, as expected. The reduction in the expression of the *galiomycin* gene following administration of the peptide seems to suggest that gH625-M could have an anti-inflammatory effect which may result in the protection of the infected larvae.

Interestingly, gH625-M treatment before the infection, significantly reduced the biofilm-associated gene expression of *HWP1* and *ALS3* particularly in co-culture, supporting the efficacy of the peptide already observed in vitro. It was previously reported that *HWP1* mutants produce a thin biofilm with less hyphae in vitro, but display serious biofilm defects in vivo, only forming yeast microcolonies, while *ALS3* mutants are able to form hyphae, but exhibit defects in biofilm formation [31,32]. Thus, the observed down-regulation of hyphal specific gene, *HWP1*, could determine a loss of physical scaffolds for yeast cell adhesion and aggregation, producing a decreased biofilm strength, integrity, and maturation. The results obtained confirm previous hypothesis on the ability of gH625-M in regulating the initial adhesion of yeast cells to surfaces, which is essential for all stages of biofilm development.

For *K. pneumoniae*, too, genes involved in biofilm formation and virulence indicated that only the pre-treatment with gH625-M before infection has a significant effect in decreasing gene activity, confirming that this peptide was able to reduce infection and biofilm formation. However, future experiments relative to differential gene expression after 4 h and 12 h, when % of viability is higher than 50%, could clarify the action of these genes involved in *G. mellonella* immune response and biofilm formation in *C. albicans* and *K. pneumoniae.*

The results obtained in this study confirm the importance of developing new strategies for dealing with polymicrobial biofilms and we foresee a novel role for membranotropic peptides such as gH625 for the inhibition of biofilm formation thanks to their physico-chemical properties. It is likely that conformational flexibility and ability to destabilize hydrophobic domains typically present in membrane bilayers makes them able to disrupt the structure of the biofilm (eradication) or interfere with biofilm formation (inhibition). In conclusion, membranotropic peptides represent an appealing strategy to further evaluate for the development of innovative therapies meant to address problems such as biofilm inhibition/eradication and resistance.

## Figures and Tables

**Table 1:** Primers used in this study.

Primer Name	Primer Sequence (5′-3′)	Melting Temperature (°C)	Amplicon Length (bp)
*G.mellonella*_*actin*_F	GGACTTGTACGCCAACACAG	60	196
*G.mellonella*_*actin*_R	CCACATCTGCTGGAATGTCG	62	
*G.mellonella*_*galiomycin*_F	GGTGCGACGAATTACACCTC	62	101
*G.mellonella_galiomycin*_R	TCGCACCAACAATTGACGTT	55	
*K.pneumoniae*_16S_F	AGCACAGAGAGCTTG	54	126
*K.pneumoniae*_16S_R	ACTTTGGTCTTGCGAC	59	
*K.pneumoniae*_*luxS*_F	ATCGACATTTCGCCAATGGG	58	157
*K.pneumoniae*_*luxS*_R	ACTGGTAGACGTTGAGCTCC	66	
*K.pneumoniae*_*mrkA*_F	ACGTCTCTAACTGCCAGGC	64	115
*K.pneumoniae*_*mrkA*_R	TAGCCCTGTTGTTTGCTGGT	66	
*C.albicans*_*actin*_F	AGCCCAATCCAAAAGAGGTATT	62	153
*C.albicans*_*actin*_R	GCTTCGGTCAACAAAACTGG	63	
*C.albicans*_*HWP1*_F	CAGCCACTGAAACACCAACT	63	135
*C.albicans*_*HWP1*_R	CAGAAGTAACAACAACAACACCAG	63	
*C.albicans*_*ALS3*_F	CTAATGCTGCTACGTATAATT	56	201
*C.albicans*_*ALS3*_R	CCTGAAATTGACATGTAGCA	58	

## Data Availability

The data presented in this study are available on request from the corresponding author.
